# Vehicle Signal Analysis Using Artificial Neural Networks for a Bridge Weigh-in-Motion System

**DOI:** 10.3390/s91007943

**Published:** 2009-10-12

**Authors:** Sungkon Kim, Jungwhee Lee, Min-Seok Park, Byung-Wan Jo

**Affiliations:** 1 Seoul National University of Technology / Seoul, Korea; E-Mail: skkim@snut.ac.kr; 2 Dankook University / Yongin-si, Gyeonggi-do, Korea; 3 Korea Expressway Corporation / Sungnam-si, Gyeonggi-do, Korea; E-Mail: BMS@ex.co.kr; 4 Hanyang University / Seoul, Korea; E-Mail: joycon@hanmail.com

**Keywords:** bridge weigh-in-motion (B-WIM), artificial neural network (ANN), cable-stayed bridge, vehicle information

## Abstract

This paper describes the procedures for development of signal analysis algorithms using artificial neural networks for Bridge Weigh-in-Motion (B-WIM) systems. Through the analysis procedure, the extraction of information concerning heavy traffic vehicles such as weight, speed, and number of axles from the time domain strain data of the B-WIM system was attempted. As one of the several possible pattern recognition techniques, an Artificial Neural Network (ANN) was employed since it could effectively include dynamic effects and bridge-vehicle interactions. A number of vehicle traveling experiments with sufficient load cases were executed on two different types of bridges, a simply supported pre-stressed concrete girder bridge and a cable-stayed bridge. Different types of WIM systems such as high-speed WIM or low-speed WIM were also utilized during the experiments for cross-checking and to validate the performance of the developed algorithms.

## 1. Introduction

The concept of road vehicle weigh-in-motion (WIM) was first introduced in the 1950s in the United States. The purpose of this system was to overcome the drawbacks of static weighing and acquiring traffic information such as weight, speed, passing lane, axle spacing, and type of vehicle without interference with the traffic flow. To achieve these objectives, various sensors are installed beneath pavement layers or on a bridge superstructure and the acquired sensor signals are analyzed and saved.

Earlier WIM systems were developed as a low-speed WIM system which can be applied for vehicles at speeds less than 20 km/h, and were mainly utilized for overweight vehicle detection. Later, high-speed WIM systems were developed to improve WIM systems, but the development suffered difficulties in attaining acceptable accuracy due to the sensitive dynamic interactions between vehicles and pavement surfaces. In Korea, a high-speed WIM system was developed by the Korea Highway Corporation, and is now operating on the Central Inland Highway for pre-selecting overweight vehicles.

The first Bridge Weigh-in-Motion (B-WIM) system can be traced back to Moses and Peters [1,2]. Initially the B-WIM system was developed with an axle detector which is installed in the pavement layer to provide information such as velocity, axle spacing and the category of the vehicle. Recently, the B-WIM system is being applied as the Free Axle Detector (FAD) or the Nothing On Road (NOR) B-WIM system [3,4].

Since most of the existing B-WIM systems are developed on the static influence line theory, the accuracy can be compromised due to dynamic behaviors or dynamic interactions between the bridges and vehicles. To solve this problem, a number of researches have been performed on measured influence line [5-7], moving force identification (MFI) that utilizes mode superposition and Tikhonov regularization [8], 2-dimmensional (MFI) [9], etc. Studies have shown, in general, good accuracy for the estimating gross vehicle weight (GVW); however the accuracy decreased for individual axle weights [10].

The application of artificial neural networks (ANN) to the B-WIM was attempted in 2003 by Gonzalez *et al.* for noise removal and calibration of the system [11], and in 2005 as a research project conducted by Korea Expressway Corporation. The purpose of the project was to develop a B-WIM system for cable-stayed bridges where it was difficult to apply the conventional influence line theory [12,13].

As a continuation of the previous research, the ANN method is additionally applied to a pre-stressed concrete girder bridge, and the results of the two applications are discussed. Unlike previously developed B-WIM algorithms, the gross vehicle weight (GVW) is calculated first and then the axle weights are calculated by distributing GVW using axle weight distribution factors (AWDFs) in this study. The ANN is utilized to calculate both GVW and axle weights sequentially.

## 2. Hardware Installation, Data Acquisition and ANN Construction

### 2.1. Description of the Bridges

**Figure 1.**
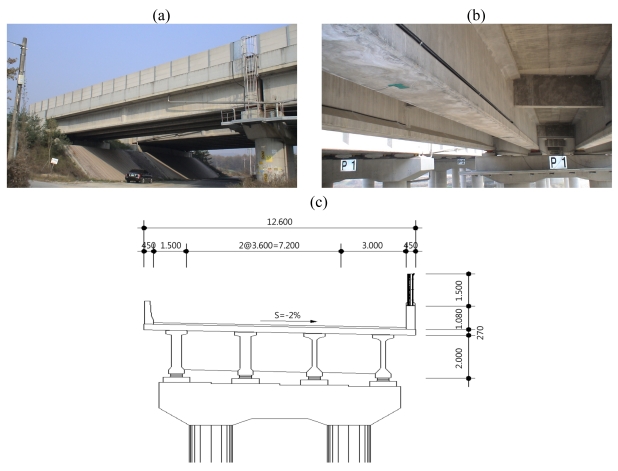
(a) Geumdang Bridge site. (b) Main girders, cross beams and a concrete deck. (c) Typical section (unit: m).

Two different types of bridges, namely a ore-stressed concrete (PSC) girder bridge and a cable-stayed bridge were employed in this study. The first bridge, a pre-stressed concrete girder bridge, is the four simple spans' part of Geumdang Bridge (30+3@40m) which is located on the Central Inland Highway in Korea. Strain gauges of the B-WIM system are installed on the 1st span of 30 m length. Geumdang Bridge consists of four PSC girders and a concrete deck of 12.6 m width which carries two traffic lanes. Figure 1 shows the Geumdang Bridge.

**Figure 2.**
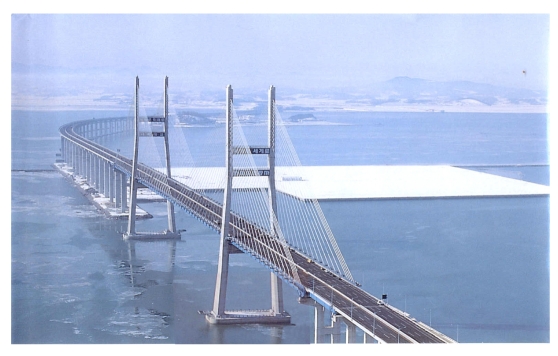
Seohae Bridge.

The second bridge, a cable-stayed bridge with a steel-concrete composite deck, is Seohae Bridge (Figure 2), which is located approximately 65 km south of Seoul. The bridge, crossing Asan Bay, is 7.31 km long and consists of a cable-stayed bridge and two different types of PSC box girder bridges. The cable-stayed bridge, which is 990 m-long, consists of a three cable-stayed spans of 200 m + 470 m + 200 m and two 60 m-long end spans of simply-supported composite girders. The B-WIM system sensors are installed on the middle of the 470 m-long center span of cable-stayed bridge.

### 2.2. Installation of Data Acquisition System

The sensors used to develop the B-WIM system are all strain gauge, and the sensors can be categorized into weight-measuring sensors and axle-detecting sensors. Weight-measuring sensors are installed on the lower surface of main girders and/or cross beams, and axle-detecting sensors are installed on the lower surface of concrete deck.

**Figure 3.**
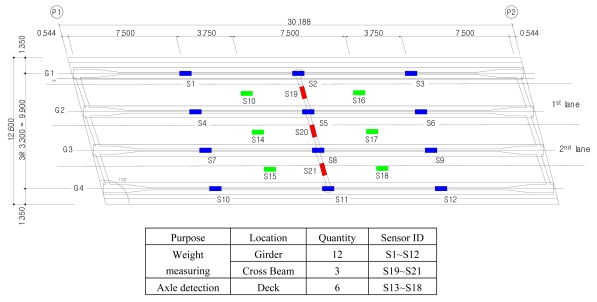
Sensor disposition of Geumdang Bridge (unit: m).

**Figure 4.**
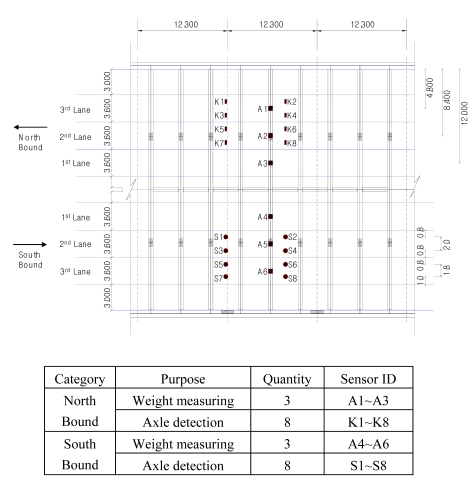
Sensor disposition of Seohae Bridge (unit: m).

In the case of the Geumdang Bridge, weight-measuring sensors were installed on both girder and cross beams to compare the performances of each case, but in Seohae Bridge, only a cross beam was instrumented with weight-measuring sensors since the strain from the main girder would not give sufficient accuracy due to its structural characteristics. Sensor dispositions are depicted in Figures 3 and 4.

### 2.3. Experimental Test Using Test Trucks

Experimental trials using test trucks which were statically measured their axle and gross weights were performed on the Geumdang Bridge and Seohae Bridge. The test trucks repeatedly traveled over a lane of the bridges at a pre-defined speed several times, and the strain signals were analyzed by the suggested B-WIM system. In case of Geumdang Bridge, three-, four- and five-axle dump trucks were employed as test trucks. The driving speed was varied from 5 km/h to 90 km/h, and the test trucks traveled 10 occasions for the 60 km/h and 90 km/h driving speed.

Similar types of test trucks such as three-, four-, and five-axle dump trucks were used on Seohae Bridge as well. The axle weights and representative test cases are depicted in Tables 1 and 2.

### 2.4. Acquisition of Random Vehicles' Data

Data of various vehicle types with widely spread weight distribution are required for the training of ANN and validation of the output weights calculated by the trained ANN. This data also must to be acquired independently from other WIM systems simultaneously. The high-speed WIM system which was developed by a research project conducted by Korean Highway Corporation, and low-speed the WIM system for overweight selection at the nearest toll-gate were utilized for Geumdang Bridge and Seohae Bridge, respectively.

### 2.5. Characteristics of B-WIM Signals

Representative B-WIM signals are illustrated below, and the characteristics of the signals are discussed in this section.

**Figure 6.**
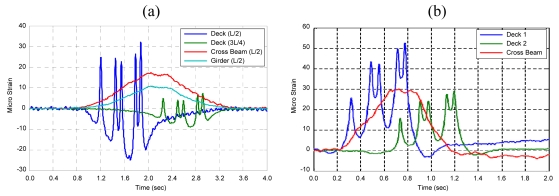
Representative B-WIM signals of (a) Geumdang Bridge. (b) Seohae Bridge.

As proven in the previous research [5], traditional axle detectors could be replaced with strain sensors installed underneath the concrete deck of PSC girder bridges such as Geumdang Bridge since appropriate strain readings could be acquired for obtaining information about number of axles, speed and axle spacings of a vehicle. Also, appropriate strain readings for calculating gross vehicle weight (GVW) or axle weights could be obtained from the main girders or cross beams. In the case of Geumdang Bridge, it could be expected that utilizing strains of cross beams could improve GVW accuracy since the strain reading of the cross beam showed a higher level than that of the main girder [Figure 6(a)].

Since Seohae Bridge is a cable-stayed bridge with a 470 m-long main span, utilizing strains of the main girder makes weight calculation difficult since the number of vehicles simultaneously existing on the considering span increased due to structural characteristics. Moreover, stay cables anchored with a regular spacing of 12.3 m make the derivation of theoretical influence line complex. Therefore, strains of cross beams were believed to be more suitable for calculation of weights for this bridge.

Typical B-WIM signals of Geumdang Bridge and Seohae Bridge are shown in Figure 6. Appropriate deck strains for replacing conventional axle detectors are observed in both bridges. The cross beam strain also shows a shape applicable for weight calculations.

### 2.6. Design of the ANN Structure

The conventional influence line theory first calculates axle weight directly from the measured signal and then the GVW is derived by summing axle weights. In this study, the contrary procedure is suggested. First, the GVW is calculated basically using strain readings of main girders and/or cross beams by GVW calculating ANN, then axle weights are resulted by multiplying GVW and axle weight distribution factors (AWDFs) which is the output of another ANN. The major input parameter of the AWDF calculating the ANN is the peak strain values of concrete deck which corresponds to each axle of a passing vehicle.

#### ANN for GVW calculation

The principal input parameters of the GVW calculating ANN are: (1) the peak strain readings of main girders and/or (2) the peak strain readings of cross beams. Six channels of strain signals which consist of three channels of main girders and three channels of cross beams were available in Geumdang Bridge. In contrast, signals only from three channels of cross beams corresponding to the direction of traveling vehicle were utilized for Seohae Bridge. Vehicle speed, summation of axle spacing and summation of peak strains of the deck were additionally included in the ANN input parameters to increase accuracy.

#### ANN for axle weight distribution factor (AWDF) calculation

An individual ANN was constructed separately for calculating axle weight distribution factors (AWDFs) which are used to calculate weight of each axle by multiplying to the resulting GVW. The input values of this ANN are peak strain values of the concrete deck corresponding to the axle of passing vehicle and axle spacings, and the output values of this ANN would be the AWDF for each axle. When AWDFs are obtained from the ANN, axle weights can be simply calculated by multiplying the GVW and AWDFs. The structure of ANN for AWDF calculation is depicted in Table 4.

## 3. GVW Calculation Using ANN

### 3.1. Training of ANN

Independent data was acquired from an adjacent WIM system (i.e., high-speed WIM or low-speed WIM for overweight selection) as mentioned previously.

**Figure 7.**
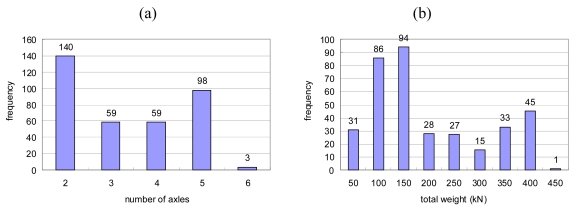
Geumdang Bridge random truck cases' histogram of (a) Number of axles. (b) Gross vehicle weight (GVW).

The acquired data set was appropriate for training and testing the ANN since the data set contains of vehicles with various numbers of axles (from 2 to 6 axles) and total weights (from 100 to 400 kN), as shown in Figure 7. A part of the data set was separated and utilized as the training set, and the remaining part was utilized as the test set.

When compared to the case of utilizing static weights, utilizing the WIM data of random trucks leads to relatively low convergence accuracy since the target values which were extracted from the WIM data contain certain portion of error from the former. However, it has advantages from the viewpoint of amount, distribution, acquisition time and cost of data when other WIM systems are available.

**Figure 8.**
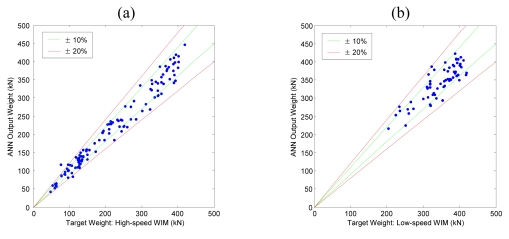
Results of training of (a) Geumdang Bridge—cross beam strain. (b) Seohae Bridge—north bound 3^rd^ lane.

Figure 8 shows comparative graph between target input values (high-speed or low-speed WIM data) and resulting output values of the training data. When an output value perfectly agrees with the target value, the corresponding data point will be located on the *y* = *x* line, and increasing error between target and output values disperses the data points from the *y* = *x* line. In Figure 8, ±10% and ±20% error lines are also plotted with green and red lines respectively.

### 3.2. Validation of Trained ANN

**Figure 9.**
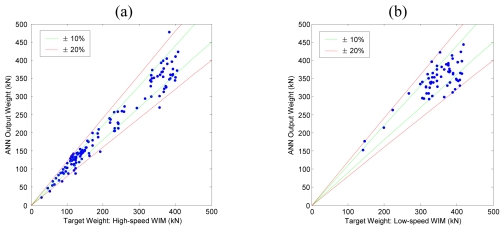
Results of validation test of (a) Geumdang Bridge—cross beam strain. (b) Seohae Bridge—north bound 3^rd^ lane.

Calculated output weights of ANN for the remaining data which were not used for training were compared to the target values as a validation test. As shown in Figure 9, both the Geumdang Bridge and Seohae Bridge cases gave similar error levels to those of the training data cases (Figure 8).

Although the error statistics can be calculated with these results, discussion on the accuracies of the systems is not appropriate since the target values are not static weights and they initially contained errors. Therefore, accuracy of the systems will be discussed later with the results of test trucks which were statically weighed and driven.

### 3.3. Comparison of Accuracies Using Experimental Data of Test Trucks

Statically pre-weighed test trucks of 3-, 4-, and 5-axles were driven repeatedly. Then the calculated dynamic weight from the suggested B-WIM systems were compared to the static weights, and finally the accuracy classes of the European WIM specification, which were established by the WAVE project were determined from statistical analysis of the relative errors between the dynamic and static weights.

**Figure 10.**
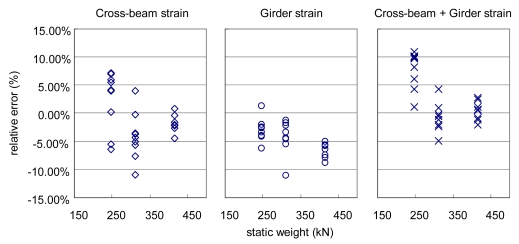
Performance comparison between ANN input parameters (Geumdang Bridge).

The resulting errors of the dynamic weights and accuracy determination results of Geumdang Bridge are presented in Figure 10 and Table 5. The applied vehicle speed was 90 km/h and the test trucks were repeatedly driven nine times. Accuracy resulting from the influence line method using girder strain readings is presented for comparison in Table 5.

Comparing the results in Table 5 to the recent research result reported by McNulty and O'Brien, the ANN method results show similar accuracy classes to the influence line method in GVW calculation, therefore ANN methods can be considered as an alternative when application of the influence line method is not suitable. In Table 6, the GVW accuracy results of Seohae Bridge are depicted.

Though a lower accuracy class than the existing low-speed WIM system resulted from the ANN method, similar accuracy classes to the Geumdang Bridge cases could be achieved.

## 4. Axle Weight Calculation Using ANN

Existing influence line methods calculate individual axle weights first, and then the gross vehicle weight (GVW) is calculated by summing up the axle weights. In contrast, the suggested ANN method calculates GVW first, and then axle weights are calculated with axle weight distribution factors (AWDFs). Data acquired on the Geumdang Bridge is utilized to compare the performance of the axle weight calculations. The structure of the ANN for axle weight calculation is as mentioned previously, and the result is compared to the influence line method in this section.

### 4.1. Training and Validation Test of ANN

The training set of the axle weight calculating ANN was prepared from the high-speed WIM system which is located near the Geumdang Bridge as in the previous case. According to the number of axles of passing vehicles, individual ANNs for 3-, 4-, and 5-axle trucks were constructed and trained using random vehicles' data. When training was completed, the validation test followed using the remaining data that were not used for training.

**Figure 11.**
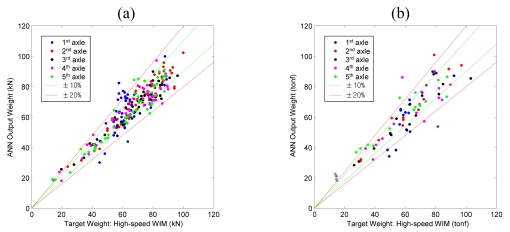
ANN construction for Geumdang Bridge (5-axle random trucks) (a) Results of training. (b) Results of validation test.

Results of training and validation test are shown in Figure 11. Since data points of training set [Figure 11(a)] and test set [Figure 11(b)] shows similar dispersion levels, it can be said that the ANN is trained properly.

### 4.2. Comparison of Accuracies Using Experimental Data of Test Trucks

Determination of the axle weight accuracy class is carried out with the results of a 10 times' repeated experimental test of pre-weighed test trucks. The speed of the vehicles was controlled to closely maintain 90 km/h. The following figure shows the results of axle weight calculation with respect to the corresponding static weights. It can be confirmed that an overall ±20% error bound is satisfied.

As the case of GVW calculation, resulting accuracy classes of axle weights are compared with the results of a recent research using influence line method [7] in Table 7 and Table 8. Since the two compared cases are not dealing with the same bridge, the accuracy class cannot be compared directly. However, considering that the span length of the bridge studied in the previous research is 15 m, and that shorter bridge will generally show higher accuracy result, it can be said that the two compared methods show similar accuracy level.

## 5. Conclusions

In this study, the applicability of artificial neural networks (ANN) is investigated for the improvement of conventional B-WIM systems so that it can be implemented on long-span bridges (such as cable-stayed bridges) where the application of influence line theory had difficulties.

The proposed algorithm mainly consists of two separately developed stages which are calculations from the gross vehicle weight (GVW) and the distribution of this GVW into individual axle weights, and ANNs for each stage. The ANN for the 1st stage calculates GVW by analyzing the dynamic strain signal measured from the main girders and/or cross beams, and the 2nd ANN calculates GVW distribution factors using peak strain values of concrete deck, axle spacings and speed of the passing vehicle. Finally, individual axle weights can simply be calculated from GVW and GVW distribution factors.

Data acquired from adjacent independent WIM systems (low-speed WIM or high-speed WIM) were utilized for the training and validation tests of the ANNs. Experimental test data from three-, four-, and five-axle trucks whose their static weights were previously measured were also utilized for the accuracy class calculation that is established by the WAVE project.

The proposed method is applied to two different types of bridges (a pre-stressed concrete girder bridge, Geumdang Bridge, and a steel-concrete composite cable stayed bridge, Seohae Bridge) and the results compared to those of the conventional method.

For the GVW calculation, the proposed ANN method and conventional influence line method show similar accuracy classes. Therefore, the ANN method can be considered as an alternative to the influence line method for long-span bridges where it cannot be applied easily.

Moreover, the ANN method results in higher accuracy class than the influence line method for axle weight calculation. Consequently, the combination of both the influence line method and the ANN method is also possible for improving the axle weight calculation accuracy of existing B-WIM systems.

## Figures and Tables

**Figure 5.**
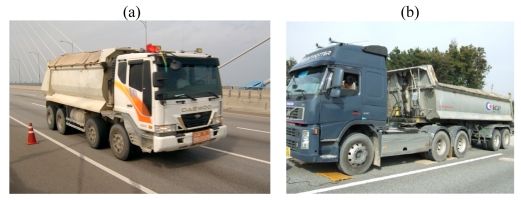
(a) Experimental test on Seohae Bridge. (b) Static weighing of test trucks for Geumdang Bridge.

**Figure 12.**
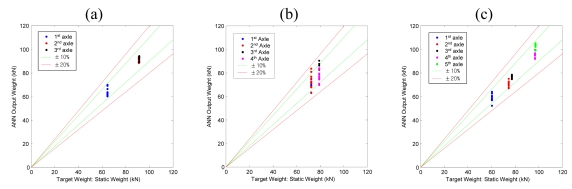
Performances of trained ANN (a) 3-axle test truck. (b) 4-axle test truck. (c) 5-axle test truck.

**Table 1.** Weight of test trucks and test cases of Geumdang Bridge.

**Type of vehicle**	**Axle weight (kN)**					**Speed (km/h)**	**No. of repetition**
	**1**	**2**	**3**	**4**	**5**		
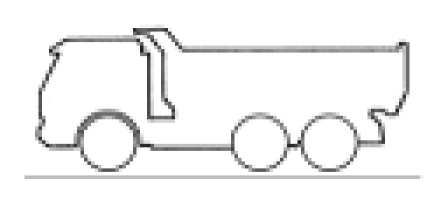	65.0	91.3	91.6	-	-	5	1
						10 ∼ 50	1
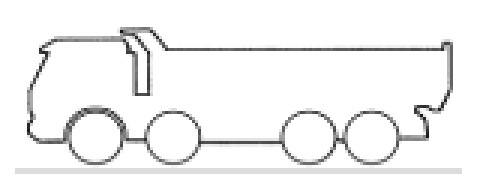	73.6	73.9	80.8	80.8	-	60	10
						70, 80	1
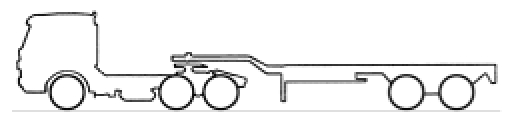	61.2	75.5	78.2	98.2	98.5	90	10

**Table 2.** Weight of test trucks and test cases of Seohae Bridge.

**Type of vehicle**	**Axle weight (kN)**					**Speed (km/h)**	**No. of repetition**
	**1**	**2**	**3**	**4**	**5**		
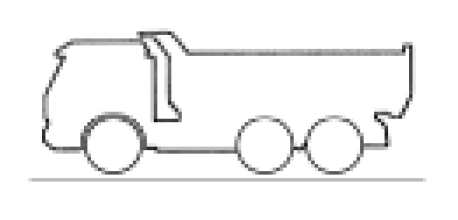	67.0	85.3	81.2	-	-	60	50
						65	10
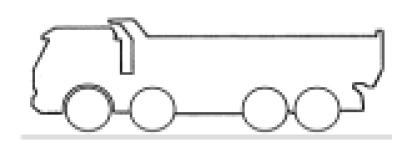	71.5	92.2	71.0	85.8	-	70	48
						75	8
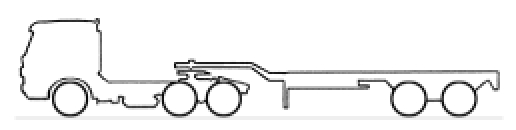	59.6	79.3	79.4	88.7	88.6	80	40

**Table 3.** Structures of ANN for GVW calculation.

	**Geumdang Bridge**	**Seohae Bridge**
Input parameters (number of values)	o Peak strain values of Cross beam (3) and/or girder (3)	
	o vehicle speed (1)	
	o Σ axle distances (1)	
	o Σ peak strain values of deck (1)	
Layer (number of node)	Input layer: 1 (6 or 9 nodes)	Input layer: 1 (6 nodes)
	Hidden layer: 2 (10 and 7 nodes)	Hidden layer: 1 (10 nodes)
	Output layer: 1 (1 node)	Output layer: 1 (1 node)
Transfer function	Pure linear – pure linear – pure linear	

**Table 4.** Structure of ANN for AWDF calculation.

Input parameters (number of values)	o Peak strain values of deck (=number of axles) o axle distances (=number of axles-1)
Layer (number of node)	Input layer: 1 (2 × {number of axles} - 1) Hidden layer: 2 (10 and 5) Output layer: 1 (number of axles)
Transfer function	Pure linear – pure linear – pure linear

**Table 5.** Accuracy results of GVW from various B-WIM algorithms (Geumdang Bridge).

**Algorithm**		**Number**	**Mean (%)**	**St.dev. (%)**	**π_0_ (%)**	**Class**	**δ (%)**	**π (%)**	**Class retained**
ANN	Cross-Beam	26	-1.62	4.52	94.9	C(15)	15.0	99.0	**C(15)**
	Girder	26	-4.99	2.38	94.9	B(10)	10.0	94.9	**B(10)**
	Cross-Beam & Girder	24	1.56	3.77	94.7	B(10)	10.0	95.2	**B(10)**

**Table 6.** Accuracy results of GVW from B-WIM and Low-speed WIM (Seohae Bridge).

**Algorithm**	**Number**	**Mean (%)**	**St.dev. (%)**	**π_0_ (%)**	**Class**	**δ (%)**	**π (%)**	**Class retained**
ANN (Cross-beam)	25	0.58	5.46	94.7	C(15)	15.0	97.1	**C(15)**
Low-speed WIM	33	-3.82	2.71	95.5	B(10)	10.0	96.8	**B(10)**

**Table 7.** Accuracy results of axle weights from ANN method.

**Criterion**	**Number**	**Mean (%)**	**St.dev. (%)**	**π_0_ (%)**	**Class**	**δ (%)**	**δ_min_ (%)**	**π (%)**	**Class retained**
single axle	40	-1.45	6.20	95.6	C(15)	20.0	15.2	99.3	**C(15)**
group of axles	40	0.41	3.81	95.6	B+(7)	10.0	9.2	97.3	
axle of a group	80	0.40	4.99	96.6	B+(7)	14.0	11.9	98.8	

**Table 8.** Accuracy results of axle weights from influence line method [7]

**Criterion**	**Number**	**Mean (%)**	**St.dev. (%)**	**π_0_ (%)**	**Class**	**δ (%)**	**δ_min_ (%)**	**π (%)**	**Class retained**
single axle	188	-1.31	7.27	93.7	B(10)	15	14.8	94.0	**B(10)**
group of axles	239	-0.18	5.26	93.9	B(10)	13	10.6	98.0	
